# The Roles of Alpha-Momorcharin and Jasmonic Acid in Modulating the Response of *Momordica charantia* to *Cucumber Mosaic Virus*

**DOI:** 10.3389/fmicb.2016.01796

**Published:** 2016-11-09

**Authors:** Ting Yang, Yao Meng, Li-Juan Chen, Hong-Hui Lin, De-Hui Xi

**Affiliations:** ^1^Ministry of Education Key Laboratory for Bio-Resource and Eco-Environment, College of Life Science, Sichuan UniversityChengdu, China; ^2^School of Medical Laboratory Science, Chengdu Medical CollegeChengdu, China

**Keywords:** alpha-momorcharin, jasmonic acid, salicylic acid, reactive oxygen species, *Cucumber mosaic virus*, Momordica charantia

## Abstract

Alpha-momorcharin (α-MMC) is a type-I ribosome inactivating protein with a molecular weight of 29 kDa that is found in *Momordica charantia*, and has been shown to be effective against a broad range of human viruses as well as having anti-tumor activities. However, the role of endogenous α-MMC under viral infection and the mechanism of the anti-viral activities of α-MMC in plants are still unknown. To study the effect of α-MMC on plant viral defense and how α-MMC increases plant resistance to virus, the *M. charantia*–*cucumber mosaic virus* (CMV) interaction system was investigated. The results showed that the α-MMC level was positively correlated with the resistance of *M. charantia* to CMV. α-MMC treatment could alleviate photosystem damage and enhance the ratio of glutathione/glutathione disulfide in *M. charantia* under CMV infection. The relationship of α-MMC and defense related phytohormones, and their roles in plant defense were further investigated. α-MMC treatment led to a significant increase of jasmonic acid (JA) and vice versa, while there was no obvious relevance between salicylic acid and α-MMC. In addition, reactive oxygen species (ROS) were induced in α-MMC-pretreated plants, in a similar way to the ROS burst in JA-pretreated plants. The production of ROS in both ibuprofen (JA inhibitor) and (α-MMC+ibuprofen)-pretreated plants was reduced markedly, leading to a greater susceptibility of *M. charantia* to CMV. Our results indicate that the anti-viral activities of α-MMC in *M. charantia* may be accomplished through the JA related signaling pathway.

## Introduction

Plants and pathogens have been engaged in an ongoing game of one-upmanship for millions of years. Plant viruses utilize multiple strategies to eliminate plant defenses and then promote their replication in host plants. To survive, plants have evolved a range of defense mechanisms, such as hormone-mediated signaling pathways and gene silencing pathways, to increase their defenses against pathogen attack (Gaffney et al., 1993; Pieterse et al., 2009; Chen et al., 2013). Hormones such as JA, SA, and abscisic acid (ABA) are primarily involved in the plant defense responses in plant–virus interactions (Zhu et al., 2014; Alazem and Lin, 2015). These pathways can be cooperative, or antagonistic through a complex network. Thus, plants can adjust the level of cross-talk to maintain an effective defense under pathogen attack (Koornneef and Pieterse, 2008; Robert-Seilaniantz et al., 2011). Although, plants have evolved a range of defense mechanisms to increase their defenses against pathogen attack, agricultural crops worldwide still suffer from a vast array of diseases, which cause tremendous yield and quality losses (Culbreath et al., 2003; Rodoni, 2009). It is known that some plants possess specific metabolic pathways to synthesize the number of valuable proteins that can be used for the prevention and treatment of diseases (Calixto, 2000). For example, in recent years it has been reported that plant genes encoding ribosomal inactivating proteins (RIPs) could affect the disease tolerance and resistance of plants (Huang et al., 2007; Dowd et al., 2012).

Ribosomal inactivating proteins are toxic *N*-glycosidases that function by irreversibly inhibiting protein synthesis through the removal of one or more adenine residues from 28S ribosomal RNA (Stirpe and Battelli, 2006; Puri et al., 2012). It has been reported that many RIPs have anti-viral, anti-cancer, deoxyribonuclease, and antibacterial activities (Stirpe, 2004; Kaur et al., 2011). RIPs could enhance the resistance of plants against viruses, bacterium, and fungi *in vitro* and *in vivo*. For example, it has been clearly demonstrated that the expression of α-MMC in transgenic rice plants can prevent rice blast (Qian et al., 2014). α-MMC is a member of the type I family of RIPs, with a molecular weight of approximately 29 kDa, and is derived from *Momordica charantia.* It has important biological properties in animals, including DNA hydrolase, rRNA *N*-glycosidases, anti-HIV, antibacterial, and antiviral activities (Fang et al., 2012; Pan et al., 2014). Our previous study showed that foliar spraying of α-MMC on tobacco plants exhibited multiple antiviral activities against phytopathogenic viruses and antifungal activity (Zhu et al., 2013). In addition, several reports describing the relationship between RIPs and abiotic stress responses, such as drought, salinity, and heavy metal contamination, have been published (Jiang et al., 2012; Wang et al., 2016). Moreover, numerous studies have shown the involvement of α-MMC in bacterial defense responses (Lodge et al., 1993; Yuan et al., 2002).

Plant response to various stresses is frequently associated with the generation of ROS (Zhang et al., 2001; Baxter et al., 2014). For a long time, ROS were considered to be harmful and to cause damage to plants. Notably, several lines of evidence proved that high levels of ROS caused cell death (Choi et al., 2007; Yang et al., 2011). For example, brassinosteroids-induced abiotic stress resistance was reduced after H_2_O_2_ treatment in *cucumber* plants (Wei et al., 2015). However, other studies have suggested that ROS, especially H_2_O_2_ encoded by *RBOH* genes, have a positive effect on plant responses to biotic and abiotic stresses (Xia et al., 2011; Deng et al., 2016a). Many studies have shown that low levels of ROS act as a defense signal in plants. For example, in *Nicotiana benthamiana*, ROS generation was shown to inhibit virus replication (Deng et al., 2016a). In another study, H_2_O_2_ was shown to decrease the susceptibility of *zucchini* to CMV (Tao et al., 2015). Hence, the role of ROS remains uncertain in plant–virus interactions.

*Cucumber mosaic virus* is considered to be one of the world’s most important viruses due to its wide range of hosts. It is also one of the few viruses that can infect *M. charantia* both naturally and experimentally. α-MMC is a natural secondary metabolite encoded by the endogenous gene of *M. charantia*. Therefore, the *M. charantia*–CMV interaction system could be an excellent model for investigating α-MMC induced host responses to viral infection. In this study, a chemical treatment demonstrated that α-MMC could inhibit virus replication by inducing a plant defense that was dependent on JA. Moreover, α-MMC played a positive role in plant resistance and the activation of JA. Additionally, JA in turn influenced the accumulation of α-MMC in *M. charantia*–CMV interactions. Notably, our results showed that ROS acted as a second messenger in α-MMC and JA-induced CMV defense responses.

## Materials and Methods

### Plant Materials and Growth Conditions

*Momordica charantia* plants were grown in a temperature-controlled growth room under a 16 h-light/8 h-dark cycle (100 mol m^-2^ s^-1^) at 20–25°C. Experiments were performed at the stage when the second leaves of *M. charantia* plants were fully expanded.

### Chemical Treatments and Virus Inoculation

The α-MMC was extracted from seeds of *M. charantia* according to the method of Bian et al. (2010). JA, SA, ibuprofen, and ABT were purchased from Sigma-Aldrich (St. Louis, MO, USA). The hormone and inhibitor solutions were prepared in water containing 0.02% (vol/vol) Tween 20. The following concentrations of chemicals were used: JA (100 μM), SA (100 μM), α-MMC (0.5 mg/ml), ibuprofen (100 μM), and ABT (1 nM). Distilled water containing 0.02% (vol/vol) Tween 20 was used as a control treatment. For the α-MMC+ ABT+SA treatment, seedlings were pretreated with ABT, then 12 h later were treated with α-MMC for 24 h, and were then exposed to the virus before being treated with SA 3 days later. To investigate the roles of hormones in the resistance, leaves were pretreated with ibuprofen or ABT, and 12 h later these plants were exposed to the virus. For the ibuprofen+JA treatment, plants were pretreated with ibuprofen, then 12 h later were infected with CMV before being treated with JA three days later.

In infection experiments, the chemicals were sprayed 24 h before virus inoculation. CMV was maintained in an aqueous suspension of 0.02 M sodium phosphate buffer (PBS, pH 7.0) at 4°C. The inoculation with virus was implemented as described previously (Deng et al., 2016b). PBS buffer was used as a control. All experiments were repeated three times.

### Superoxide, H_2_O_2_ Staining, and Determinations

Superoxide and H_2_O_2_ staining were detected with NBT and the H_2_O_2_ fluorescence probe H_2_DCFDA (Sigma-Aldrich). *M. charantia* leaves were vacuum infiltrated with NBT (0.5 mg/mL) solutions for 2 h. Leaves were then decolorized in boiling ethanol (90%) for 15 min. The method used for H_2_O_2_ fluorescence probe staining was described by Deng et al. (2016b). The Amplex red hydrogen peroxide/peroxidase assay kit (Invitrogen, Waltham, MA, USA) was used to determine H_2_O_2_ accumulation.

### Damage Estimation

Electrolyte leakage was measured as described by Yang et al. (2004). After measuring the conductivity of the fresh leaves, they were boiled for 60 min to achieve 100% electrolyte leakage. Lipid peroxidation was estimated by measuring the MDA as previously described (Velikova et al., 2000). The lipid peroxides were expressed as MDA content.

### Determination of Antioxidant Enzymes

For the enzyme assays, 0.3 g of leaf material was ground with 3 mL ice-cold 25 mM Hepes buffer (pH 7.8) containing 0.2 Mm EDTA, 2 mM ascorbate and 2% PVP. The homogenates were centrifuged at 4°C for 20 min at 12,000 *g* and the resulting supernatants were used for the determination of enzymatic activity. SOD, CAT, APX, and POD activities were assayed as described by Wang et al. (2011). ASA, DHA, GSH, and GSSG were extracted and determined as described by Xu et al. (2012).

### Analysis of Chlorophyll Fluorescence

Chlorophyll fluorescence was determined with an imaging pulse amplitude modulated fluorometer (IMAG-MINI; Heinz Walz, Effeltrich, Germany). For the measurement of Fv/Fm, plants were first dark adapted for 30 min. Minimal fluorescence (Fo) was measured during the weak measuring pulses, and maximal fluorescence (Fm) was measured by a 0.8 s pulse of light at about 4000 l mol m^-2^ s^-1^. An actinic light source was then applied to obtain a steady-state fluorescence yield (Fs), after which a second saturation pulse was applied for 0.7 s to obtain the light-adapted maximum fluorescence (Fm_0_). Fv/Fm and ΦPSII were calculated as Fm – Fo/Fm and (Fm_0_ – Fs)/Fm_0_, respectively.

### JA and SA Determination

*Momordica charantia* plants were grown in soil and inoculated following the different treatments and systemic leaves were used for hormone determination. SA and JA were quantified by high-performance liquid chromatography–mass spectrometry (HPLC–MS) from crude plant extracts according to the method of Zhu et al. (2013). As internal standards, 2-hydroxybenzoic acid-[^2^H_6_] (d_6_-SA) was obtained from Sigma-Aldrich, and dihydrojasmonic acid (H_2_JA) was obtained from OlChemim (Olomouc, Czech Republic) (Zhu et al., 2014).

### RNA Extraction and Quantitative Real-Time Polymerase Chain Reaction (qRT-PCR)

The total cellular RNA was extracted using a previously described method (Wang et al., 2010). The RNA content was calculated by measuring the absorbance value taken at 260 nm. All RNA samples were treated with DNase I before PCR. The qRT-PCR analysis was performed with the primers shown in Supplementary Table S1. Relative quantitation of the target gene expression level was performed using the comparative Ct. Three technical replicates were performed for each experiment, including at least three independent plants. 18S RNA was used as an internal control.

### Protein Extraction and Western Blot Analysis

The total proteins were extracted with extraction buffer (50 mM Tris-Cl, pH 6.8, 5% mercaptoethanol, 10% glycerol, 4% SDS, and 4 Murea) in an ice bath. The protein concentrations were determined through the Bradford method, using bovine serum albumin as the standard (Xi et al., 2007). Western blot analysis was performed according to the protocol described Xi et al. (2007).

### Statistical Analysis

The data were expressed as the mean ± SD and statistically analyzed using a one-way analysis of variance (ANOVA). A difference was considered to be statistically significant when *P* < 0.05.

## Results

### Spatiotemporal Expression of α-MMC in *M. charantia*

**FIGURE 1:**
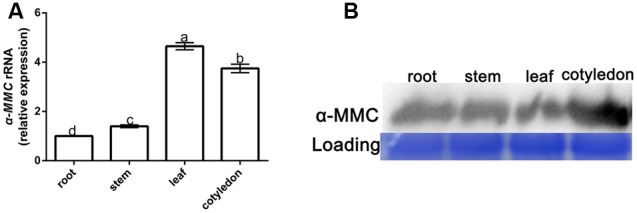
**Spatiotemporal expression of α-MMC in *Momordica charantia.* (A)** A qRT- PCR analysis of α-MMC accumulation levels in the root, stem, leaf, and cotyledon, respectively. 18S RNA was used as an internal control. Bars represent the mean and standard deviation of values obtained from three biological replicates per genotype and time point. Significant differences (*P* < 0.05) are denoted by different lower case letters. **(B)** Western blot analysis of α-MMC in the root, stem, leaf, and cotyledon. Rubisco proteins were used as loading controls and were stained by Coomassie Brilliant Blue.

The qRT-PCR and Western blot analysis were used to detect the expression of α-MMC in different parts of the *M. charantia* plants. Seven-day old *M. charantia* plants were divided into four parts: root, stem, leaf, and cotyledon. The qRT-PCR results with α-MMC specific primers indicated that the highest transcription level of α-MMC was in the leaf. The gene expression level of α-MMC in cotyledons was slightly reduced compared with the leaf. The lowest transcription of α-MMC was in the root (five times lower than the leaf and about 35% lower than the stem (**Figure 1A**). As shown in **Figure 1B**, the Western blot analysis results indicated that the highest accumulation of α-MMC protein occurred in the cotyledon, with intermediate levels in the root, which confirmed the results of previous reports (Stirpe and Battelli, 2006).

### α-MMC Suppressed CMV Replication and Accumulation in Inoculated and Systemic Leaves

**FIGURE 2:**
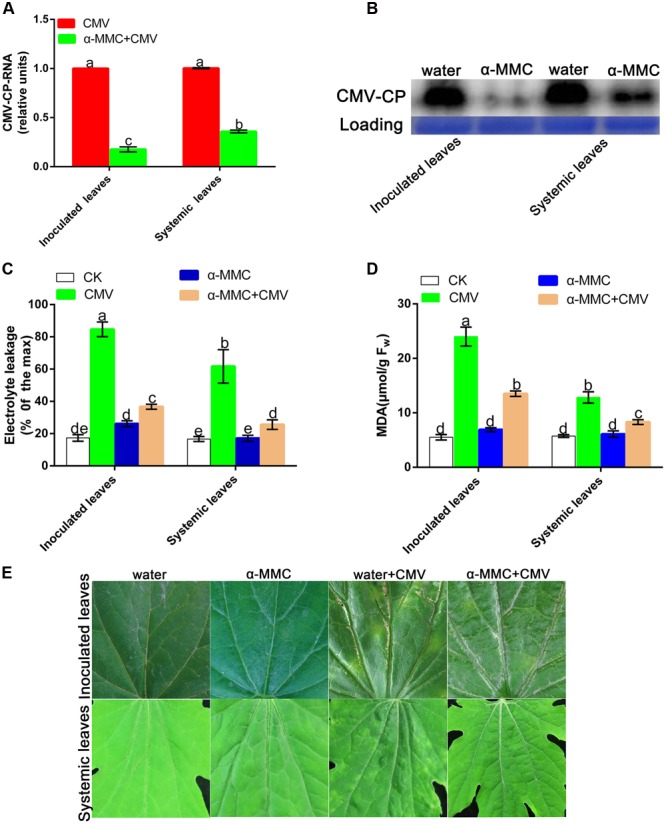
**α-MMC suppressed CMV replication and accumulation in inoculated and systemic leaves. (A)** A qRT- PCR analysis of CMV mRNA accumulation in inoculated leaves at 5 dpi and systemic leaves at 9 dpi. 18S RNA was used as an internal control. Bars represent the mean and standard deviation of values obtained from three independent biological replicates. **(B)** Western blot analysis of coat protein accumulation of CMV in inoculated leaves at 5 dpi and systemic leaves at 9 dpi. Rubisco proteins were used as loading controls and were stained by Coomassie Brilliant Blue. Changes in electrolyte leakage **(C)** and MDA content **(D)** under CMV infection. Bars represent the mean and standard deviation of values obtained from three independent biological replicates. **(E)** Phenotype of water and α-MMC-pretreated plants with or without CMV infection at 8 dpi. Experiments were repeated three times with similar results. Significant differences (*P* < 0.05) are denoted by different lowercase letters.

To investigate the role of α-MMC in the *M. charantia* defense against CMV, plants were pretreated with α-MMC and water before CMV infection and the CMV replication and accumulation levels were then detected by qRT-PCR and Western blot analysis in inoculated and newly grown leaves (systemic leaves). As shown in **Figure 2A**, the transcription level of CMV decreased significantly in α-MMC-pretreated plants compared with water-pretreated plants. The qRT-PCR result was also confirmed by Western blot analysis (**Figure 2B**). The trends of the replication and accumulation of CMV on systemic leaves and inoculated leaves were consistent. These results confirmed the role of α-MMC in the *M. charantia* defense against CMV.

The MDA content and electrolyte leakage indicated the degree of damage in plants caused by biotic stresses. To verify the damage to the plasma membrane caused by the plant virus infection, the MDA content and electrolyte leakage were measured in inoculated and systemic leaves. As shown in **Figure 2C**, water pretreated plants had more serious damage and a greater occurrence of cell-death than α-MMC pretreated plants. The change of electrolyte leakage and the MDA content in systemic and inoculated leaves were consistent (**Figure 2D**). At 8 dpi, *M. charantia* infected leaves developed strong disease symptoms characterized by yellow spots and mosaics compared with control plants. Water pretreated plants displayed more serious symptoms than α-MMC-pretreated plants (**Figure 2E**). The results indicated that α-MMC played a positive role in *M. charantia* resistance to CMV infection.

### Effects of α-MMC on the Antioxidant Capacity under CMV Inoculation

**FIGURE 3:**
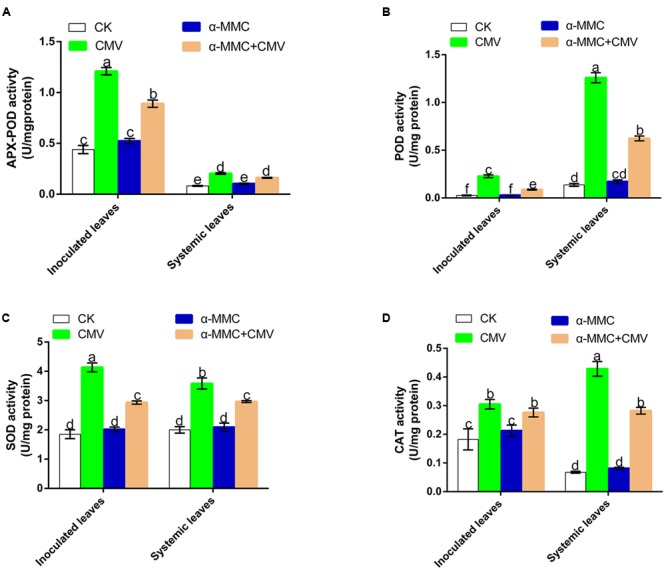
**Analysis of antioxidant enzyme activities under CMV infection at 5 dpi in inoculated and at 9 dpi in systemic leaves.** APX-POD **(A)**, POD **(B)**, SOD **(C)**, CAT **(D)**. Bars represent the mean and standard deviation of values obtained from three independent biological replicates. Experiments were repeated three times with similar results. Significant differences (*P* < 0.05) are denoted by different lowercase letters.

Activation of antioxidant capacity was important to avoid oxidative damage in plants under virus infection. To investigate whether antioxidant systems participate in an α-MMC induced CMV defense response, we examined the activity of the antioxidant enzymes SOD, CAT, POD, and APX-POD. As shown in **Figure 3**, the enzyme activities of all treated plants were elevated under CMV infection, but α-MMC pretreatment did not boost the activity of antioxidant enzymes. In contrast, the enzyme activities were lower than in plants pretreated with water under virus infection. These results suggested that α-MMC may reduce the oxidative damage in some way that is independent of antioxidant capacity.

### Effects of Exogenous α-MMC on Endogenous α-MMC and Hormone Production

**FIGURE 4:**
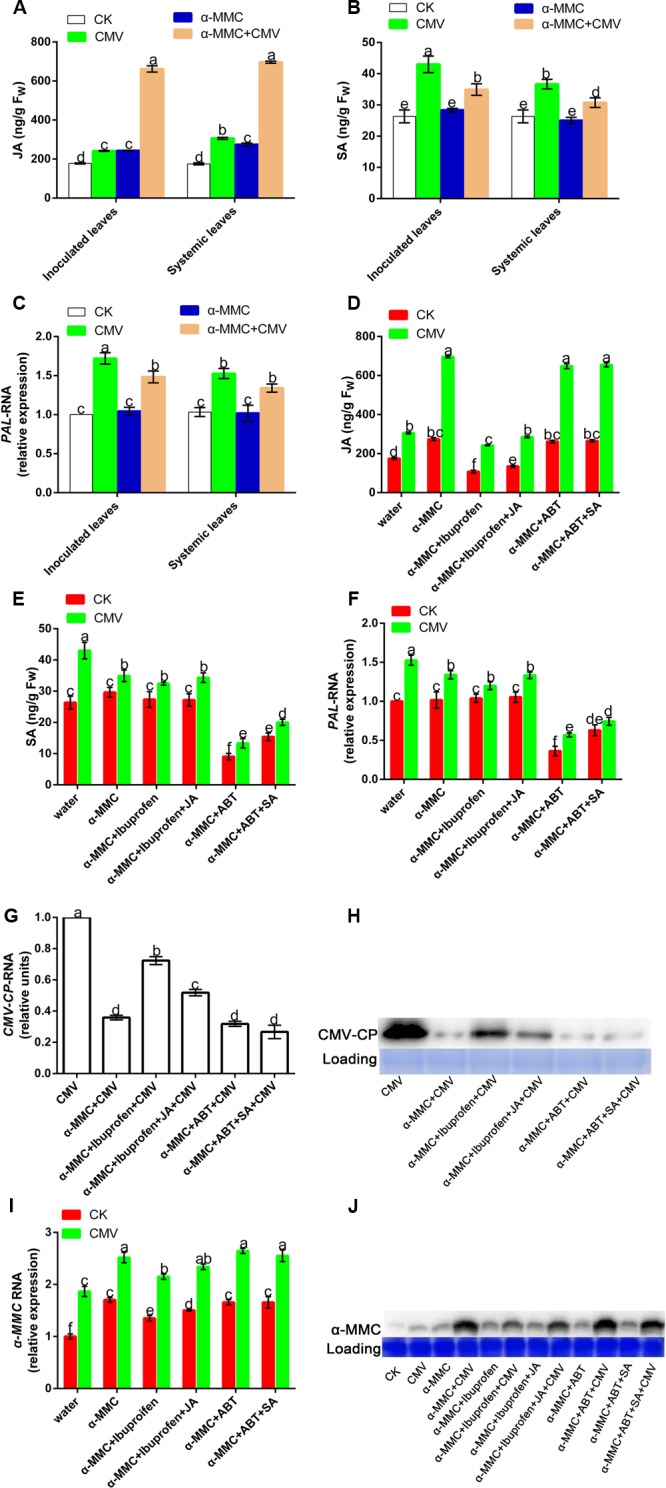
**Effects of exogenous α-MMC on endogenous α-MMC and hormone production.** JA and SA accumulation **(A,B)** in the water and α-MMC-pretreated plants with or without CMV infection, and the expression of the SA biosynthesis gene **(C)** at 5 dpi in inoculated leaves and 9 dpi in systemic leaves. JA and SA accumulation **(D,E)** in different pretreatments and the expression of the SA biosynthesis gene **(F)** at 9 dpi in systemic leaves. A qRT-PCR analysis of CMV and α-MMC mRNA accumulation levels **(G,I)** and Western blot analysis of the CMV coat protein and α-MMC accumulation **(H,J)** in systemic leaves collected at 9 dpi. 18S RNA was used as the internal control. Error bars represent the mean and standard deviation of values obtained from three independent biological replicates. Experiments were repeated three times with similar results. Significant differences (*P* < 0.05) are denoted by different lowercase letters.

Hormones play vital roles in plant–pathogen interactions, with SA, and JA known to be involved in defense responses. We investigated whether α-MMC-induced CMV resistance was dependent on these hormones by quantifying the levels of SA and JA in pretreated plants. As shown in **Figures 4B,C**, there was no significant difference in the accumulation of SA in control and inoculated plants, and CMV infection caused little variation in SA levels. However, α-MMC pretreatment and CMV infection could induce JA accumulation. Furthermore, the JA content increased more in α-MMC-pretreated plants than in water-pretreated plants under virus infection (**Figure 4A**). Based on these results, we speculated that α-MMC induced a CMV defense that was dependent on JA content, but not on SA content.

To further investigate the roles of hormones in limiting CMV infectivity, we used a JA inhibitor (ibuprofen) and an SA inhibitor (ABT) to determine their functions in CMV infection (Zhu et al., 2006; Wang et al., 2012). To test the effect of the inhibitors, we evaluated hormone contents and the transcription of *PAL*, which plays a crucial role in SA synthesis in pretreated plants. As shown in **Figures 4D–F**, these inhibitors could effectively inhibit hormone biosynthesis. CMV replication and accumulation increased in ibuprofen-pretreated plants compared with plants that were only pretreated with α-MMC. However, there was no significant difference in ABT-pretreated plants (**Figures 4G,H**). These results also proved that the α-MMC induced virus defense depends on JA, but not SA.

Because α-MMC exhibits anti-inflammatory and anti-viral effects in animals, we further investigated its expression in plants after chemical treatment and CMV inoculation. To identify the molecular mechanisms involved in the α-MMC response to CMV, we examined the transcription of the α-MMC synthesis gene and its protein accumulation. The qRT-PCR and Western blot results showed that foliar applications of α-MMC induced the up-regulation of endogenous α-MMC (**Figures 4I,J**). Transcription and accumulation of α-MMC were greater in α-MMC-pretreated plants than in water-pretreated plants under CMV infection, while all of them were up-regulated. As shown in **Figure 4G**, CMV accumulation in α-MMC+ ibuprofen-pretreated plants was increased compared with α-MMC-pretreated plants, but was less than in water-pretreated plants. The expression of α-MMC was inversely proportional to CMV replication and accumulation (**Figures 4G,H**). Taken together, these results suggest that α-MMC was a positive regulator in α-MMC-induced viral resistance in *M. charantia*.

### Involvement of ROS in α-MMC-Induced CMV Defense

**FIGURE 5:**
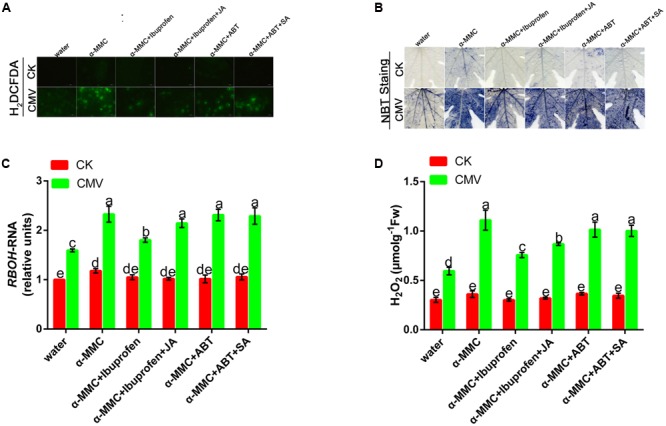
**Involvement of ROS in α-MMC-induced CMV defense.** H_2_DCFDA- **(A)** and NBT-stained **(B)** control or CMV inoculated *M. charantia* leaves pretreated with water, α-MMC, α-MMC+Ibuprofen, α-MMC+Ibuprofen+JA, α-MMC+ABT, or α-MMC +ABT+SA at 5 dpi in systemic leaves. **(C)** A qRT-PCR analysis of the *RBOH* gene at 5 dpi. 18S RNA was used as the internal control. **(D)** H_2_O_2_ level in control or CMV inoculated leaves determined at 5 dpi. Error bars represent the mean and standard deviation of values obtained from three independent biological replicates. Experiments were repeated three times with similar results. Significant differences (*P* < 0.05) are denoted by different lowercase letters.

To determine the possible role of ROS in α-MMC-induced virus resistance in *M. charantia*, we attempted to detect the *in situ* accumulation of superoxide (O^2-^) and H_2_O_2_ using NBT and H_2_DCF-DA staining procedures, respectively. The results showed that both O^2-^ and H_2_O_2_ increased in α-MMC-pretreated leaves compared with water-pretreated leaves (**Figures 5A,B**). We further detected H_2_O_2_ levels in these leaves. Similarly, in α-MMC-pretreated plants, the H_2_O_2_ content was significantly higher than in water-pretreated plants infected with CMV, which was consistent with the *RBOH* gene (**Figures 5C,D**). The results suggested that α-MMC could induce an ROS burst in response to CMV infection. Importantly, α-MMC-induced ROS accumulation was again largely inhibited by ibuprofen, but not by ABT.

### JA Plays a Positive Role in Photosystem Protection in α-MMC-Induced CMV Resistance

**FIGURE 6:**
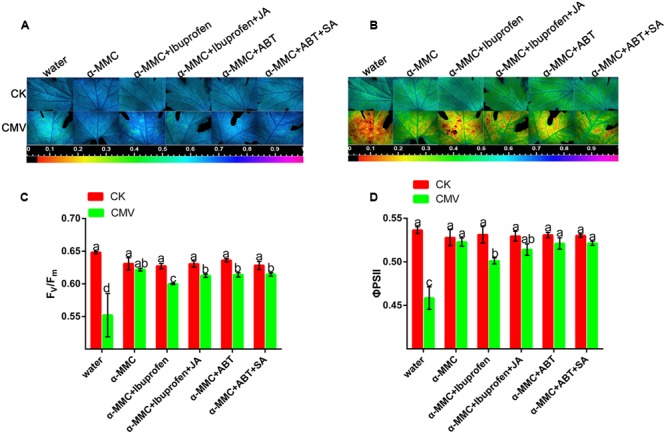
**Jasmonic acid plays a positive role in photosystem protection in α-MMC-induced CMV resistance. (A,B)** Images of Fv/Fm and ΦPSII at 9 dpi. Four plants were used for each treatment and a picture of one representative plant is shown. **(C,D)** Average values for the respective chlorophyll fluorescence image replicates. Error bars represent the mean and standard deviation of values obtained from three independent biological replicates. Experiments were repeated three times with similar results. Significant differences (*P* < 0.05) are denoted by different lowercase letters.

Pathogens and environmental stress can disturb the photochemistry of photosystem II (PSII) and induce a photoprotection mechanism. F_V_/Fm and ΦPSII are indicators of PSII photochemical activity. To determine the roles of the hormone in the α-MMC-induced defense response to CMV, we investigated the effects of ibuprofen, JA inhibitor and ABT, SA inhibitor on the α-MMC-induced resistance to CMV challenge. As shown in **Figures 6A,C**, the Fv/Fm of water-pretreated plants was significantly lower than in α-MMC-pretreated plants. In contrast, non-photochemicalexciton quenching (NPQ) was higher in water-pretreated plants, but lower in α-MMC-pretreated plants under CMV infection at 9 dpi (**Figures 6B,D**). The lower Fv/Fm indicated a decline of photosynthesis, while the higher NPQ implied some degree of photo-damage suffered by the plant. Taken together, the results indicated that α-MMC could protect the photo-system of plants under virus infection. However, α-MMC-induced resistance to photo-oxidative stress was largely inhibited if the plants were pretreated with ibuprofen, but were not influenced by ABT (**Figures 6C,D**). The application of JA almost rescued the decrease in stress resistance due to ibuprofen. These results showed that JA played a positive role in photo-system protection in α-MMC-induced CMV resistance.

### JA Improves Plant Resistance under CMV Infection

**FIGURE 7:**
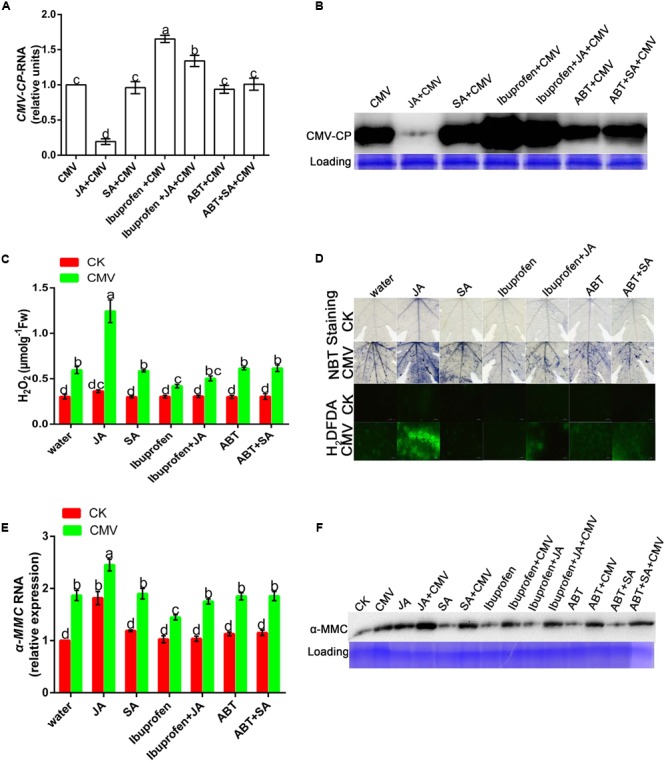
**Jasmonic acid improves plant resistance under CMV infection. (A,E)** A qRT-PCR analysis of CMV and hormone-induced α-MMC mRNA accumulation in systemic leaves at 9 dpi. 18S RNA was used as an internal control. Bars represent the mean and standard deviation of values obtained from three independent biological replicates. **(B,F)** Western blot analysis of coat protein accumulation of CMV and α-MMC-protein accumulation in systemic leaves at 9 dpi. Rubisco proteins were used as loading controls and were stained by Coomassie Brilliant Blue. **(C)** H_2_O_2_ content. Error bars represent the mean and standard deviation of values obtained from three independent biological replicates. Significant differences (*P* < 0.05) are denoted by different lowercase letters. **(D)** Superoxide contents were detected by NBT staining and H_2_O_2_ levels were detected by H_2_DCFDA staining at 5 dpi in water-, hormone- or hormone inhibitor pretreated plants with or without CMV infection. Experiments were repeated three times with similar results.

Salicylic acid and JA are important natural hormones that have a function in plant resistance against virus infection. To explore the effects of JA and SA on plant resistance under CMV infection, we used hormones and relevant inhibitors in this experiment (**Supplementary Figure S2**). There was less accumulation of CMV in JA-pretreated plants than in water-pretreated plants (**Figures 7A,B**). However, the level of viral replication was significantly higher in ibuprofen-pretreated plants than in water-pretreated plants, but this level of viral replication could be reduced by applying JA. As shown in **Figure 7B**, there was no significant difference in the level of virus accumulation among SA, SA inhibitor, and water-pretreated plants. These results showed that plant resistance could be induced by JA but not SA.

From the above results, we concluded that ROS participated in the α-MMC-induced CMV defense. To further investigate whether JA activated plant innate immunity related to ROS, NBT, and H_2_DCF-DA staining were used in this experiment. The results indicated that JA pretreatment led to a substantial increase in the production of ROS compared with water pretreatment, but this could be inhibited by ibuprofen (**Figures 7C,D**). All of the results suggested that ROS were involved in JA-induced CMV resistance.

## Discussion

The use of α-MMC to treat human diseases such as tumors, HIV, and fungal infections has been well-studied in the past (Bian et al., 2010; Puri et al., 2012). However, studies of the role of α-MMC in plant virus resistance have rarely been reported. Therefore, the role of α-MMC in plant defense and the mechanisms involved are not well-understood. This study provides an insight into the characterization of the role of the α-MMC-mediated defense response in plants using *M. charantia* and the CMV interaction system. We revealed that α-MMC, JA, and ROS played important roles in α-MMC-mediated CMV defense in *M. charantia.*

### Spatial Differences in the Expression of α-MMC in *M. charantia*

The α-MMC level was associated with disease resistance in plants, but its gene transcription and protein accumulation were not the same in different organs. Therefore, the spatiotemporal expression of α-MMC was investigated in *M. charantia* by qRT-PCR and protein hybridization. Western blot analysis revealed that a higher accumulation of α-MMC existed in the cotyledon and root, which was consistent with the results of previous research (Kaur et al., 2012). The highest level of transcription of α-MMC appeared in the leaf and the least was in the root. Based on these results, we hypothesized that because the root and stem were lignified and the cotyledon was a vestigial organ, their level of gene transcription was lower than in the leaf.

### α-MMC-Induced CMV Defense Was Dependent on JA Levels in *M. charantia*

Plants are endowed with an innate immune system, and phytohormones such as SA and JA have been reported to play an important role in plant immunity. In this study, the JA content increased in α-MMC-pretreated plants under virus infection, along with a higher viral resistance. This result confirmed the previous reports that hormones contribute to plant biotic stresses resistance (Alazem and Lin, 2015; Tao et al., 2015). We further revealed that only JA, and not SA, was involved in the α-MMC-induced defense in *M. charantia*. When JA biosynthesis was inhibited by its inhibitor, virus accumulation increased (**Figures 4G,H**). In contrast, the SA content and the expression of *PAL* showed little variation among the different treatments infected by CMV, while ABT pretreatment did not affect the resistance induced by α-MMC (**Figures 4B,G**). These results suggest that the response of JA accumulation to virus infection was activated in α-MMC-pretreated plants, therefore they displayed an enhanced insensitivity to CMV infection.

### JA Content Was Increased by α-MMC Pretreatment and Could also Enhance α-MMC Accumulation in the *M. charantia*–CMV Interaction System

In this study, we tried not only to reveal the potential mechanisms of α-MMC’s function in CMV resistance, but also to investigate the relationship between α-MMC-induced resistance and phytohormone mediated defense pathways. Interestingly, under the exogenous application of JA the accumulation of CMV was significantly suppressed, and under the exogenous application of ibuprofen the replication of CMV was increased. However, the expression of the virus was almost unaffected by the SA or ABT treatment compared with the control (**Figures 7A,B**). These results showed that virus infection was inhibited by JA, but not SA. In the α-MMC-induced resistance, α-MMC could enhance the JA content under CMV infection (**Figure 4A**). In hormone-pretreated plants, JA could induce the expression of α-MMC, but SA could not (**Figures 7E,F**). Taken together, our results indicate that α-MMC is involved in JA-induced CMV resistance in *M. charantia*, while SA was unlikely to be involved in JA and α-MMC activation.

As described above, JA was involved in α-MMC-induced CMV resistance in *M. charantia* and played a vital role in the response to CMV. Therefore, we tested the relationship of α-MMC and hormones in the response of *M. charantia* to CMV. To achieve this, JA, ibuprofen, SA, ABT, and α-MMC were used. The results showed that α-MMC expression increased in JA-pretreated plants, but decreased in the ibuprofen pretreated group (**Figures 7E,F**). In α-MMC-pretreated plants, JA accumulation also increased. The accumulation of SA was not affected by α-MMC, and it could not induce the expression of α-MMC (**Figures 4A,B** and **7E,F**). These data indicated that α-MMC could not only affect the JA content, but JA could also regulate the expression and accumulation of α-MMC. However, α-MMC and SA could not be induced by each other.

### ROS Act as a Second Messenger in α-MMC and JA-Induced CMV Defense

Most forms of biotic or abiotic stress disrupt the metabolic balance of cells, resulting in the enhanced production of ROS. The roles of ROS in incompatible plant–virus interactions have been studied previously (Moeder et al., 2005; Király et al., 2008). However, ROS involvement in compatible plant–virus interactions is still controversial (Fu et al., 2010; Shi et al., 2012). Compared with other pretreated plants, in α-MMC or JA-pretreated plants, the levels of ROS were the highest, but plant damage was the least (**Figures 5**, **6**, and **7B,C**). Virus replication and accumulation were inversely related to ROS in the JA and α-MMC-induced defense (**Figures 4G**, **5**, and **7A,C**). These results suggest that ROS acted as a second messenger to mediate the JA and α-MMC signal during the induction of stress resistance. Although ROS are considered to be an important cellular signal, they can be cytotoxic. However, there was no obvious up-regulation of the activities of several antioxidant enzymes compared with the control. Conversely, there was a significant up-regulation in CMV-infected plants (**Figure 3**). Interestingly, the ratios of GSH/GSSH and ASA/DHA were increased in α-MMC-pretreated plants compared with water-pretreated plants, but they were decreased in plants pretreated with α-MMC+ ibuprofen under CMV infection (**Supplementary Figure S1**). The results showed that α-MMC may depend on reducing substances to avoid oxidative damage, but not antioxidant enzymes.

In summary, the results presented in this study provide evidence that α-MMC-induced plant resistance depends on the JA content under CMV infection, while the SA pathway did not display a relationship with the α-MMC-regulated viral defense response. ROS acted as a second messenger in the plant defense response to CMV infection. Our study presents new evidence that in the *M. charantia*–CMV interaction system, the molecular resistance mechanism induced by α-MMC was similar to that induced by JA. Thus, our results have revealed the novel roles of α-MMC and JA in plants against CMV infection and clarified the relationships between JA, ROS, SA, and α-MMC during CMV infection in *M. charantia*, although an understanding of the detailed mechanism needs further investigation.

## Author Contributions

D-HX and TY contributed to the experimental design of the study. TY and YM performed the experiments, data analysis, and drafted the manuscript. L-JC and H-HL read and corrected the manuscript.

## Conflict of Interest Statement

The authors declare that the research was conducted in the absence of any commercial or financial relationships that could be construed as a potential conflict of interest.

## Supplementary Material

FIGURE S1 **Changes in the activities of GSH/GSSH (A)** and ASA/DHA **(B)** in *M. charantia* under CMV infection at 9 dpi. The JA pathway was inhibited by ibuprofen or the SA pathway was inhibited by ABT pre-treatment in these α-MMC-treated plants. Error bars represent the mean and standard deviation of values obtained from three independent biological replicates. Experiments were repeated three times with similar results. Significant differences (*P* < 0.05) are denoted by different lowercase letters.

FIGURE S2 **Detection of the JA content (A)** and SA content **(B)** in hormone-pretreated and hormone inhibitor-pretreated *M. charantia* at 9 dpi. Error bars represent the mean and standard deviation of values obtained from three independent biological replicates. Experiments were repeated three times with similar results. Significant differences (*P* < 0.05) are denoted by different lowercase letters.

## Abbreviations

α-MMC: alpha-momorcharin
ABT: 1-aminobenzotriazole
ASA: ascorbic acid
CAT: catalase
CMV: *cucumber mosaic virus*
DHA: dehydroascorbic acid
dpi: days post-inoculation
EDTA: ethylenediaminetetraacetic acid
Fv/Fm: the maximal quantum efficiency of PSII
GSH: glutathione
GSSG: glutathione disulfide
H_2_DCFDA: 2′,7′-dichlorofluorescein diacetate
H_2_O_2_,: hydrogen peroxide
JA: jasmonic acid
MDA: malondialdehyde
NBT: nitro blue tetrazolium
O^2-^: situ accumulation of superoxide
*PAL*,: phenylalanine ammonia-lyase
POD: peroxidase
PVP: polyvinylpyrrolidone
qRT-PCR: quantitative real-time polymerase chain reaction
*RBOH*,: respiratory burst oxidase homolog
ROS: reactive oxygen species
SA: salicylic acid
SOD: superoxide dismutase
ΦPSII: quantum efficiency of PSII
