# Supplementary figures and images for: The Roles of Alpha-Momorcharin and Jasmonic Acid in Modulating the Response of Momordica charantia to Cucumber Mosaic Virus
Source: Front Microbiol. 2016 Nov 9;7:1796. doi: 10.3389/fmicb.2016.01796 (PMC5101195; doi:10.3389/fmicb.2016.01796)

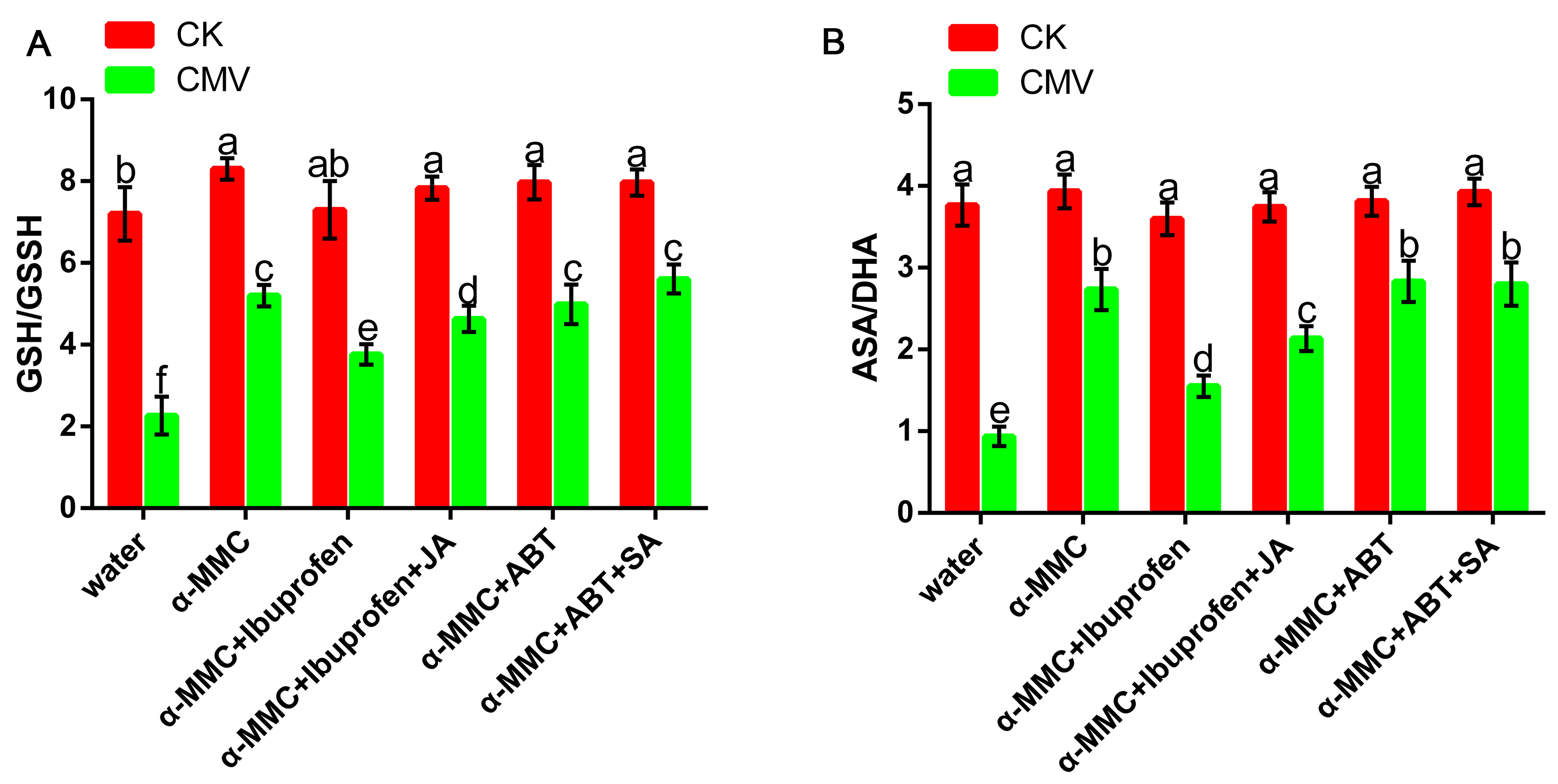

Supplement: FIGURE S1 — Changes in the activities of GSH/GSSH (A) and ASA/DHA (B) in M. charantia under CMV infection at 9 dpi. The JA pathway was inhibited by ibuprofen or the SA pathway was inhibited by ABT pre-treatment in these α-MMC-treated plants. Error bars represent the mean and standard deviation of values obtained from three independent biological replicates. Experiments were repeated three times with similar results. Significant differences (P < 0.05) are denoted by different lowercase letters. [file Image_1.JPEG]

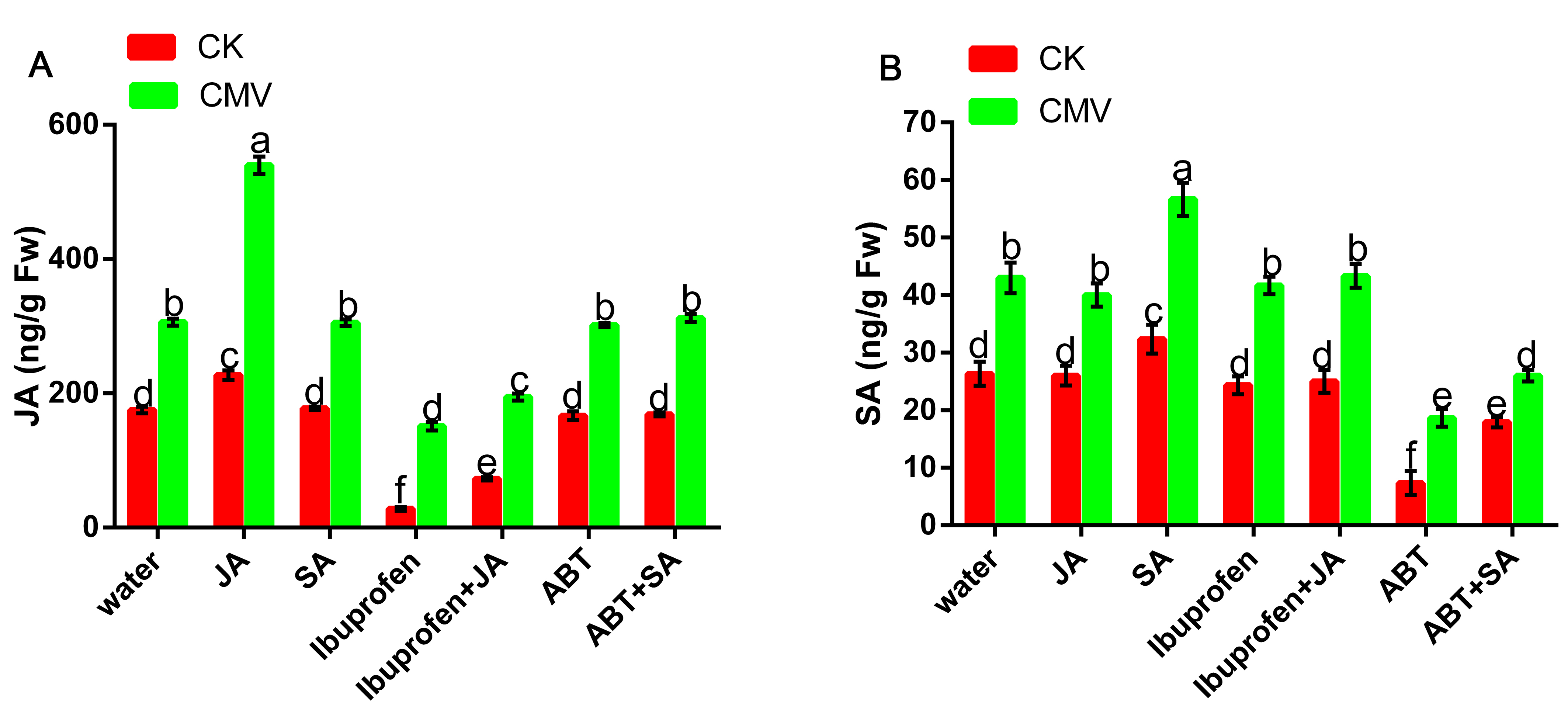

Supplement: FIGURE S2 — Detection of the JA content (A) and SA content (B) in hormone-pretreated and hormone inhibitor-pretreated M. charantia at 9 dpi. Error bars represent the mean and standard deviation of values obtained from three independent biological replicates. Experiments were repeated three times with similar results. Significant differences (P < 0.05) are denoted by different lowercase letters. [file Image_2.JPEG]
