# Supplementary material for: The Roles of Alpha-Momorcharin and Jasmonic Acid in Modulating the Response of Momordica charantia to Cucumber Mosaic Virus
Source: Front Microbiol. 2016 Nov 9;7:1796. doi: 10.3389/fmicb.2016.01796 (PMC5101195; doi:10.3389/fmicb.2016.01796)
Supplement: Supplementary file 3 [file Table_1.DOCX]

**Supplementary Table 1**. List of primers used in this study

| **Gene** | **Accession** | **F-primer** | **R-primer** |
| --- | --- | --- | --- |
| *18sRNA* | XR-001762667 | AGAAACGGCTACCACATCCA | CCAACCCAAGGTCCAACTAC |
| *PAL* | XM-004143308 | TTTGCGAGATATTGTAGCT | GTCCCTTGATTGAAGTGAT |
| *α-MMC* | X57682 | TCTTACTTCCTTCCGTTTCA | CACCATCTTTAGGGCATTCT |
| *RBOH* | XM-004135565 | AAGGTTGCTGTTTATCC | AATGGTCTTGAGTTGGG |
| *CMV*-CP | EF079893 | TGTTGTGTTTTTCTCTTT | TACTAACTCATCCGTCTC |
